# Novel Workflows for Separate Isolation of Pathogen RNA or DNA from Wastewater: Detection by Innovative and Conventional qPCR

**DOI:** 10.21769/BioProtoc.5189

**Published:** 2025-02-20

**Authors:** Kristina M. Babler, Helena M. Solo-Gabriele, Mark E. Sharkey, Ayaaz Amirali

**Affiliations:** 1 Department of Chemical, Environmental and Materials Engineering, University of Miami, Coral Gables, FL, USA; 2 Department of Medicine, University of Miami Miller School of Medicine, Miami, FL, USA

**Keywords:** Wastewater, Pathogen detection, Nucleic acids, Bead beating, Membrane filtration

## Abstract

Wastewater-based surveillance (WBS) can provide a wealth of information regarding the health status of communities from measurements of nucleic acids found in wastewater. Processing workflows for WBS typically include sample collection, a primary concentration step, and lysis of the microbes to release nucleic acids, followed by nucleic acid purification and molecular-based quantification. This manuscript provides workflows from beginning to end with an emphasis on filtration-based concentration approaches coupled with specific lysis and nucleic acid extraction processes. Here, two WBS processing approaches are presented, one focusing on RNA-specific pathogens and the other focused on DNA-specific pathogens found within wastewater: 1) The RNA-specific approach, employed for analyzing RNA viruses like severe acute respiratory syndrome coronavirus-2 (SARS-CoV-2) couples electronegative filtration of wastewater with the placement of the filter within a lysis buffer followed by direct RNA extraction. 2) The DNA-specific approach, employed for analyzing DNA pathogens like *Candida auris*, uses size selection membranes during filtration, subsequently followed by a lysis buffer, bead-beating, and DNA extraction. Separate workflows for RNA versus DNA isolations have the advantage of improving the detection of the target pathogen. A novel aspect of the RNA-specific workflow is the direct extraction of nucleic acids from filter lysates, which shows enhanced recoveries, whereas the DNA-specific approach requires bead beating prior to extraction. Novelty is also provided in a new qPCR approach called Volcano 2nd Generation (V2G), which uses a polymerase capable of using RNA as a template, bypassing the reverse transcriptase step normally required for qPCR.

Key features

• Membrane filtration approaches for concentrating suspended solids from wastewater. After concentration, workflows are optimized for separate recovery of RNA and DNA.

• Unique polymerase utilized to perform qPCR analysis, foregoing reverse transcription, for RNA.

• Sample products for use with other molecular techniques (e.g., sequencing) as workflow approaches generate high-quality, concentrated nucleic acid extracts with minimal inhibitors.

• Validated through COVID-19 surveillance where >1,000 samples of wastewater and >3,000 filter concentrates produced from these samples have been created and analyzed, with published results.

**This complete protocol was used in:** J Biomol Tech (2023), DOI: 10.7171/3fc1f5fe.dfa8d906

## Graphical overview


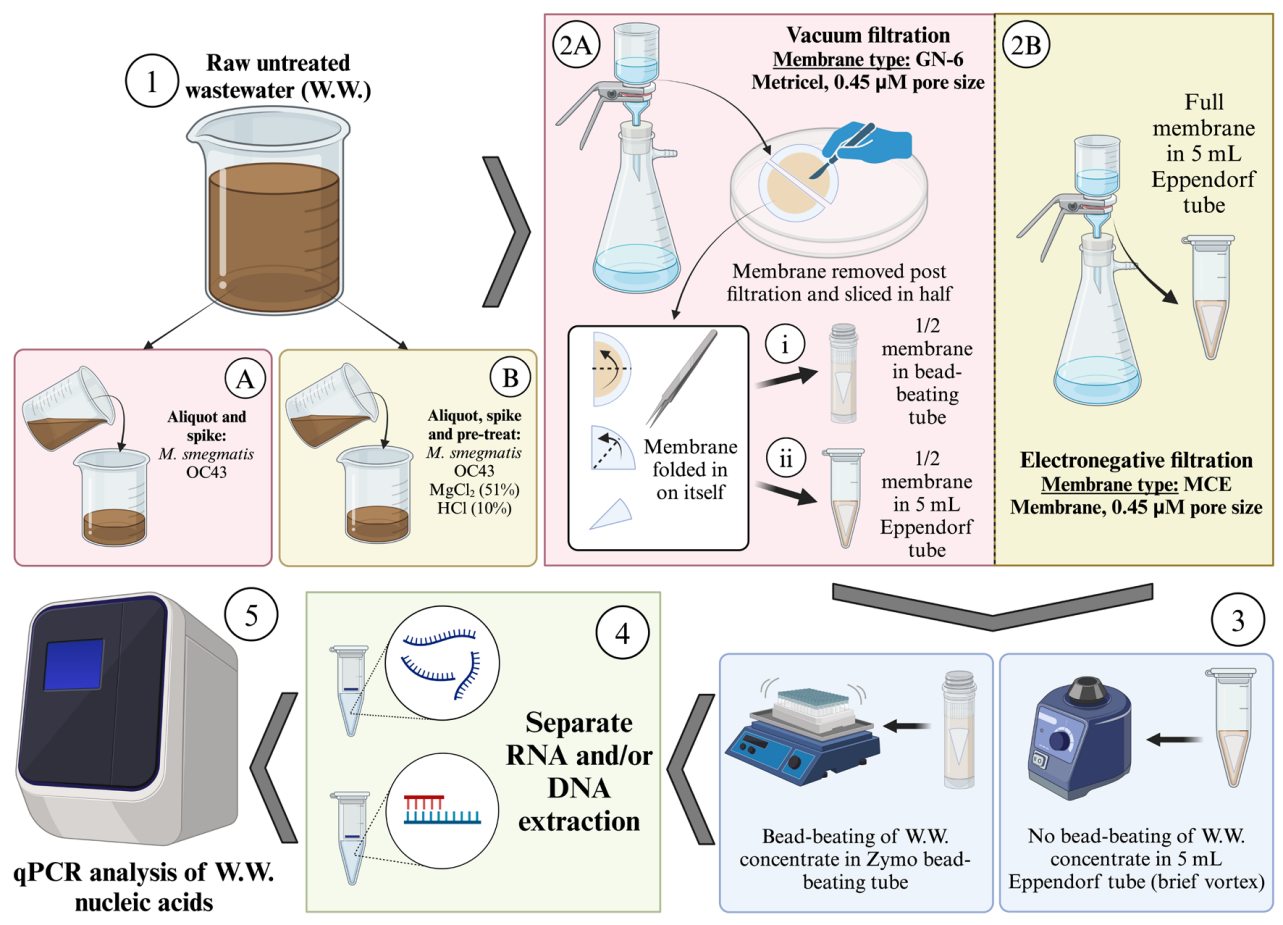


**Laboratory workflow of sample preparation**

## Background

The use of wastewater for surveying the health of communities has been a growing application in the field of public health. Analyzing environmental samples like wastewater can inform researchers of current infectious disease spread or the general health of communities. More specifically, wastewater can capture spatial and temporal trends of pollutants, drug abuse in communities, and antimicrobial resistance genes [1]. Recent wastewater-based surveillance (WBS) literature has emphasized not only WBS’s rapid development but also shed light on the importance of method selection prior to experimentation. The paper by Pecson et al. [2] is a primary example of such literature, where 32 participating laboratories at the start of the COVID-19 pandemic cooperated to compare strategies for pathogen surveillance from wastewater. Their focus was on reproducibility, sensitivity, and the impact that “other method steps” have on the recovery of viruses like SARS-CoV-2. Since the publication of Pecson et al. [2], many researchers have initiated WBS programs and have focused on comparing specific methods; a general theme has been to expand WBS’s application for SARS-CoV-2 detection to other pathogens of interest to expand WBS as a tool for public health [3–11,12,13].

Although methods are merging toward the isolation of total nucleic acids (RNA and DNA simultaneously) using the same extraction procedure, studies have shown [2] that optimal recoveries require different extraction procedures. Here, we propose two different concentration-plus-extraction strategies—one for the recovery of RNA and another for the recovery of DNA, both optimized for wastewater. The assessment of samples with the Volcano 2nd Generation (V2G) qPCR assay adds an additional uniqueness to this procedure due to the equal performance of V2G to conventional RT-qPCR [6] while providing a decrease in sample analysis time. While filtration-based concentration is not a novel concept in and of itself, its use, coupled with sample type, membrane selection, filtration protocol, lysis method, and downstream molecular techniques, is unique and adjustable for specific research needs. For example, varying volumes of wastewater can be used depending on the composition of each sample (turbidity, suspended solids concentration, etc.). Additionally, different membrane types and pore sizes can be utilized to fit the specific application. The filtration approach can be optimized for the capture of viruses by charge or for larger microbes by size exclusion. This includes pathogens like *Candida auris*, a growing health concern in many communities [14–16]. The use of bead beating, especially coupled with filtration approaches, is also advantageous as it increases the likelihood of detecting pathogens with cell membranes like *C. auris* [16] from wastewater. However, bead beating is not recommended for the recovery of RNA viruses. Bead beating can limit the detection of RNA viruses due to heat and mechanical agitation, which can lead to the degradation of some nucleic acids if not optimized prior to experimentation [3]; it also extracts ribosomal RNA from bacteria and higher forms of microbes, confounding the RNA signal from viruses. The inclusion of chemicals like HCl for the recovery of viruses by charge, although recommended in this protocol, could also impact recovery, as seen in Babler et al. [3]. Regarding limitations, filtration-based workflows require sterilization of equipment unless disposable options are favored, so they can be costly to initiate. Moreover, the processes presented here from start to finish are designed to be completed manually as opposed to magnetic bead concentration using robotic approaches [18], so laboratory capacity and personnel bandwidth also play into the ability to use these methods if many samples are expected to be analyzed. To expand these protocols to evaluate pathogens beyond SARS-CoV-2 and *C. auris*, optimization is recommended to ensure that recoveries of the intended pathogen are suitable for downstream applications. These methods, as they have been optimized for wastewater, may not be generalizable for other sample types.

## Materials and reagents

**Reagents**

**Electronegative filtration and RNA extraction workflow: specific materials and reagents**

1. Hydrochloric acid 10% (Spectrum Chemical MFG Corp., catalog number: HY105)

2. pH standards 4, 7, 10 (Cole-Parmer, catalog number: UX-05942-10)

3. Magnesium chloride, MgCl_2_, 51% w/v aqueous solution (RICCA Chemical Company, catalog number: 4470)

4. Zymo QuickRNA Viral Kit (Zymo Research, catalog number: R1034/1035)

5. V2G buffer (MyPols, catalog number: 8100)

6. V2G polymerase (MyPols, catalog number: 8400M)

7. Rox (Thermo Fisher Scientific, catalog number: 12223012)

8. dNTPs (Thermo Fisher Scientific, catalog number: R0193)

9. Platinum Taq Antibody (TaKaRa, catalog number: 9002A)

**Vacuum filtration and DNA extraction workflow: specific materials and reagents**

1. ZymoBIOMICS DNA Miniprep kit (Zymo Research, catalog number: D4300)

2. TaqMan Fast Universal PCR master mix (2×) (Thermo Fisher, catalog number: 4352042)

**Materials and reagents used for both workflows**

1. Vero Cells [American Type Culture Collection (ATCC), catalog number: CCL-81]

2. Luria Bertani (LB) Broth (Thermo Fisher Scientific, catalog number: 10855001)

3. LB agar plates (Thermo Fisher Scientific, catalog number: 22700025)

4. Tween 80 (Millipore Sigma, catalog number: P1754-25ML)

5. 100 mg/mL ampicillin (Millipore Sigma, catalog number: A5354-10ML)

6. *Mycobacterium smegmatis mc^2^155* (NIH AIDS Reagent Program, catalog number: 2195)

7. Beta coronavirus 1, strain OC43 (ATCC, catalog number: VR-1558)

8. RPMI media (Thermo Fisher Scientific, catalog number: 12633012)

9. Wastewater [collected in pre-labeled pre-sterilized bottles (500 mL capacity) containing 0.5 mL of sterile 100 g/L sodium thiosulfate; details of bottles and reagents below]

10. 1× DNA/RNA shield (Zymo Research, catalog number: R1100-250)

11. OneStep PCR Inhibitor Removal kit (Zymo Research, catalog number: D6030)

12. qPCR primers (synthesized by Sigma-Aldrich)

13. qPCR probes (synthesized by Integrated DNA Technologies)

14. Nuclease-free water (Fisher Scientific, catalog number: AM9930)

15. 99.5% isopropanol (Thermo Fisher Scientific, catalog number: 149320050)

16. 70% ethanol (Fisher Scientific, catalog number: BP82031GAL)

17. 100% ethanol (Fisher Scientific, catalog number: BP2818500)

18. Sodium thiosulfate (GrowingLabs, catalog number: LC250001)

19. Clorox bleach (concentrated)

**Laboratory supplies**

**Electronegative filtration and RNA extraction workflow: specific laboratory supplies**

1. MCE membrane filter, 47 mm, 0.45 μm pore size (Millipore Sigma, catalog number: HAWP04700)

2. Magnetic stir bars [12 mm (L) × 3 mm (D)] (VWR, catalog number: 58948-091)

3. DI water (Thermo Fisher Scientific, catalog number: 15230001)

4. Glass dropper (Fisher Scientific, catalog number: 14-955-502)

5. 5 mL Eppendorf Tubes (sterile) (Eppendorf, catalog number: 0030119460)

**Vacuum filtration and DNA extraction workflow: specific laboratory supplies**

1. Pall GN-6 Metricel MCE membrane disc filters, 47 mm, 0.45 μm pore size (New Star Environmental, catalog number: 66278)

2. 2 mL ZR BashingBead lysis tubes (0.1 & 0.5 mm) (Zymo Research, catalog number: S6012-50)

3. Polystyrene, disposable, sterile Petri dishes (Carolina, catalog number: 741248)

4. Disposable sterile scalpels #10 (Surgical Supply Service, catalog number: 75745)

**Laboratory supplies used for both workflows**

1. Biohazard waste benchtop container (1.4 L) (VWR, catalog number: 11214-708)

2. Vactrap 2 vacuum trap system for aspiration and vacuum protection (VWR, catalog number: 76207-602)

3. Pall magnetic filter funnels, 47 mm [Weber Scientific, catalog number: EF8452B (300 mL), EF8452F (500 mL)]

4. Clamps and chains to hold sample collection bottles used to collect samples from sewer holes in the field [available from any hardware store. Hose clamps large enough to attached to bottles (MIAHART, 6-inch hose clamp adjustable 304 stainless steel duct clamps worm gear), 1/8-inch chain hooks with locking connector (BNYZWOT, D-shape, twist lock), and lightweight utility chain are recommended (4every SUS304). The chain hooks are used to connect the hose clamps holding the bottles to two chains, one on each side of the bottle]

5. Strainer/colander for field (Bradshaw Home, catalog number: 72115); the strainer should have 2–3 mm diameter openings, kitchen type strainer will work

6. Funnel for field (Walmart, catalog number: HTFF-2020); the funnel should be wide at the top and narrow enough at the bottom to fit easily into the sample collection bottles

7. 5-gallon heavy-duty plastic buckets to capture spillage when pouring wastewater samples (Lowe’s, catalog number: 954434)

8. 2 L sterile plastic wide-mouth collection bottles (Thermo Fisher Scientific, catalog number: 2120-0005 if disposable, or Thermo Fisher Scientific, catalog number: 2121-0005 for reuse via autoclaving)

9. 500 mL sterile plastic collection bottles (VWR, catalog number: 16060-012 if disposable, or VWR, catalog number: 16060-012 for reuse via autoclaving); a 500 mL volume line marked on the bottle using a paint pen

10. 1 L sterile wide-mouth collection bottles (VWR, catalog number: 16060-014 if disposable, or VWR, catalog number: 16060-014 for reuse via autoclaving)

11. Kimwipes (Grainger, catalog number: 36VC06)

12. Graduated cylinders made of polypropylene for reuse by autoclaving (100 mL) (Carolina, catalog number: 721603)

13. Spade smooth tip forceps (AmScope, catalog number: TW-487)

14. PYREX griffin beakers (100 mL) (Millipore Sigma, catalog number: CLS1000100-12EA)

15. P5000 pipette (Gilson, catalog number: F144066)

16. P5000 pipette tips (Thermo Fisher Scientific, catalog number: 94052550)

17. P1000 pipette (Gilson, catalog number: F144059M)

18. P1000 filtered pipette tips (IBIS Scientific, catalog number: M-1000-9FC)

19. P200 Pipette (Gilson, catalog number: F144058M)

20. P200 filtered pipette tips (IBIS Scientific, catalog number: M-0200-9FC)

21. P100 pipette (Gilson, catalog number: F144057M)

22. P100 filtered pipette tips (IBIS Scientific, catalog number: M-0100-9FC)

23. P20 pipette (Gilson, catalog number: F144056M)

24. P20 filtered pipette tips (IBIS Scientific, catalog number: M-0020-9FC)

25. P10 pipette (Gilson, catalog number: F144055M)

26. P10 pipette tips (IBIS Scientific, catalog number: M-0010-9FC)

27. Tube racks (Eppendorf, 1.5 mL and 2 mL, catalog number: 0030119819; 5 mL and 15 mL, catalog number: 0030119827)

28. Autoclave bins (Fisher Scientific, catalog number: 13-359-20B)

29. T175 cell culture flasks (Fisher Scientific, catalog number: 10-126-34)

30. Serological pipettor (Pipette.com, catalog number: DP-501R)

31. 10 mL serological pipettes (Fisher Scientific, catalog number: NC9868325)

32. 50 mL conical tubes (Fisher Scientific, catalog number: 14-432-22)

33. 96-well plates (Bio-Rad, catalog number: HSP9601)

34. Bio-Rad microseal “B” seal (Bio-Rad, catalog number: MSB1001)

35. 1.5 mL microcentrifuge tubes (Fisher Scientific, catalog number: 05-408-129)

36. Labels for all tubes (Brady, catalog number: THT-59-7425-2-SC)

37. Freezer storage boxes (81 slot: Alkali Scientific, catalog number: FB2CC-81; 25 slot: Eppendorf, catalog number: 0030140532)

38. Laboratory coats (Fisher Scientific, catalog number: 19-181-594)

39. Disposable laboratory coats for field (Dupont Tyvek 400, catalog number: TY212S)

40. Gloves (VWR, catalog number: 76582-340)

41. Eye protection (Fisher Scientific, catalog number: 19-039-590)

42. (Optional) respiratory protection (Breatheze KN95 disposable face masks, Amazon)

43. Laboratory data sheets on clipboard with pen (created depending on lab needs using Microsoft Word or Excel)

44. Permanent paint pen (ULINE, catalog number: S-20622)

## Equipment

**Electronegative filtration and RNA extraction workflow: specific equipment**

1. pH Meter (Orion Star) (Thermo Fisher Scientific, catalog number: STARA2110)

2. Magnetic stir plate (GrowingLabs, catalog number: H4000-S)

3. Vortex Genie 2 (Scientific Industries, Inc., catalog number: SI-0236)

**Vacuum filtration and DNA extraction workflow: specific equipment**

1. BeadRuptor 12 Instrument (Omni International)

**Equipment used for both workflows**

1. YSI probe (Xylem YSI ProDSS, catalog number: 626870-1); for measuring basic water quality of samples. Recommend adding probes for pH (#626904), water temperature and salinity (#626902), turbidity (#626901), and dissolved oxygen (#626900)

2. Clean water reservoir with hand pump to rinse of equipment after sample collection (Itisll, portable garden pump sprayer, Size: 2 Gal_Brasswand, ULINE, catalog number: S-20860)

3. (Optional) Refrigerated autosampler (HACH, catalog number: AS950 fitted with an IO9000 for flow proportional sampling) or non-refrigerated autosampler (Teledyne ISCO, catalog number: ISCO 6712, fitted with a 2150 area velocity meter for flow paced sampling; otherwise, use time paced). Refrigerated with flow measurement preferred

4. BSL-2 hood

5. Whatman vacuum manifold (LabFilterz, catalog number: 10498761)

6. MyFuge centrifuge (5 mL capacity) (Grayline Medical, catalog number: C1005)

7. Eppendorf centrifuge (2 mL capacity) (Eppendorf, catalog number: 5420000245)

8. Benchtop centrifuge (Southern Labware, catalog number: C1008-B)

9. Bio-Rad CFX connect RealTime qPCR instrument (Bio-Rad Laboratories, catalog number: 1855201)

10. Plate spinner (GrowingLabs, catalog number: C2000-115V)

11. Synergy BioTek plate reader (Fisher Scientific, catalog number: BTS1LASI)

12. 37 °C shaker/incubator (Fisher Scientific, catalog number: 50-195-3922)

13. Spectrophotometer (Eppendorf, model: 6135)

14. Culture plate rocker (Millipore Sigma, catalog number: Z742529-1EA)

15. Corning LSE digital dry bath heater (Fisher Scientific, catalog number: 07-203-024)

16. Igloo or Coleman cooler (purchased from any general store)

17. Access to 4 °C, -20 °C, and -80 °C refrigerators and freezers. Freezers should not include defrost cycles to facilitate the preservation of samples in the long term

18. Access to autoclave for sterilizing reusable equipment

## Software and datasets

1. BioRender (https://www.biorender.com/). The following figures were created using BioRender: Graphical overview, Babler, K. (2024) https://www.BioRender.com/q34x703

## Procedure

**A. Wastewater collection**

Wastewater can be collected as either a grab or composite sample. A discussion about the advantages and disadvantages of grab vs. composite samples in capturing the temporal and spatial variability is available in Babler et al. [11].

1. Prior to sample collection, obtain sterile collection bottles (minimum volume of 2 L for grab samples and 5 L for composite samples). The wastewater within these collection bottles will be split and transferred into containers so that a portion of the water can be analyzed in the field for basic water quality and another portion can be brought back to the laboratory for further processing (Graphical overview, Step 1).

**Figure 1.**
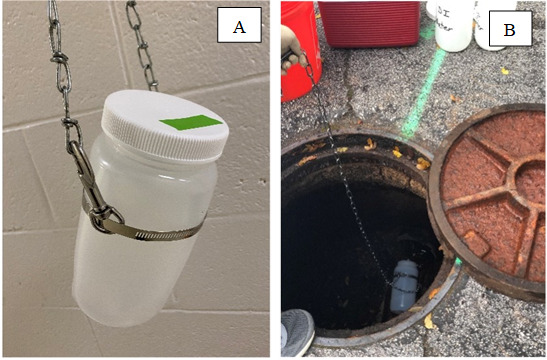
Bottle chain configuration for grab sample collection. Configuration of sampling bottle with chain connection to facilitate lowering into the sanitary sewer for grab sample collection (A). Use of bottle and chain within a sanitary sewer (B).

*Note 1: For “grab” sample type, the 2 L bottles should be fitted with a hose clamp attached to chains using two chain hooks to lower bottles down into sewer hole collection sites (Figure 1). During grab sample collection, lower the 2 L collection bottle into the sewer using its attached chains and fill with wastewater (Figure 1). Upon removal from the sewer, disinfect sample and chains by spraying with clean tap water followed by 99.5% isopropanol. Also, spray areas where wastewater may have spilled on the surface during sample collection.*

*Note 2: For “composite” sample type, the 5 L or larger bottles should be placed within an autosampler according to manufacturer’s instructions for appropriate sample collection by the autosampler. Following the completed run by the autosampler protocol, remove the bottle from the autosampler and immediately cap and disinfect the exterior collection bottle by first rinsing outside with clean water followed by spraying with 99.5% isopropanol.*

**Figure 2.**
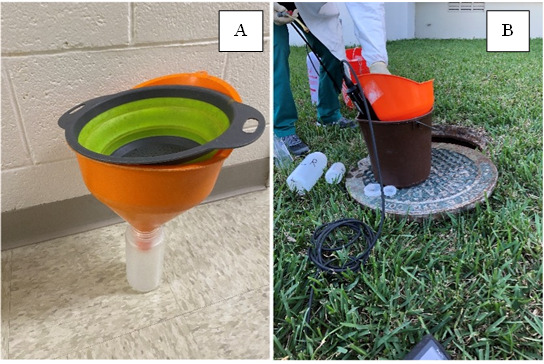
Configuration of sample collection in the field. A strainer and funnel are combined to facilitate the removal of large solids while guiding the wastewater into a bottle (A). In the field, the funnel, strainer, and bottle are placed within a bucket to capture spillage of wastewater, avoiding contamination of the surrounding ground (B). In the right panel, a water quality sonde (YSI Probe) is placed within a bottle inside the bucket, which facilitates the collection of physical-chemical water quality data in the field.

2. Upon wastewater sample collection as either grab or composites, pour collected wastewater into two separate containers. Container 1: a sterile 500 mL plastic bottle with 0.5 mL of pre-dispensed sodium thiosulfate (use 100 g/L solution of sodium thiosulfate to provide 0.1 g/L) to reduce the chlorine residual. Container 2: a rinsed 500 mL plastic container deep enough for measurement of physical-chemical water quality parameters using the YSI probe. Label Container 1 and its lid and Container 2 with a paint pen prior to transferring the wastewater, as regular water-based markers or Sharpies will be removed by alcohol sprays used to disinfect the outside of the sample bottles after wastewater sample transfer. Upon transferring wastewater from the collection bottle into each container, the samples in the collection bottles are to be homogenized (shaken), if possible, and passed through a strainer and funnel to remove large solids (Figure 2). This pouring is to occur within a 5-gallon bucket to avoid spillage on the ground. Once pouring is finished, rinse the solids from the strainer and funnel with clean water and pour captured solids back into the sewer. Disinfect the strainer and funnel with isopropanol spray. Collection bottles can either be discarded or recycled. If recycled, they are to be capped and rinsed with clean water and then sprayed with isopropanol and placed in a separate leak-proof container for transport back to the laboratory for cleaning and sterilization.

3. For Container 1 (for laboratory processing), cap with lid, rinse the outside with clean water, then spray the outside with isopropanol. Place Container on ice or with frozen ice packs within a cooler for transport to the laboratory for further processing once collected.

4. For Container 2 (for field measurements of basic water quality parameters), take water quality measurements (e.g., pH, specific conductivity, dissolved oxygen, temperature; Figure 2B) on freshly collected wastewater sample in its own reservoir utilizing a pre-disinfected and pre-calibrated YSI Probe. Record collected data upon measurement. Discard the remaining wastewater after YSI measurement back into the sewer. Rinse the YSI probe with clean water into the bucket. Discard the rinse water into the sewer. Rinse the bucket with clean water and discard the rinse back into the sewer. Disinfect YSI and bucket with isopropanol spray.

*Note: Water quality measurements are additional data that can be collected and are not necessarily a requirement to utilize this protocol. pH is the only necessary requirement (and can be completed in the laboratory) if intending to proceed to the RNA-specific filtration methods, which pretreats the sample with MgCl_2_ and HCl (lowering pH of the sample to a set range) (Section C). We do, however, recommend taking water quality parameters as environmental factors, such as water temperature, turbidity, pH, conductivity, or dissolved oxygen, that could impact pathogen recovery downstream. Therefore, having this data on a per-sample basis could be used to understand decreased pathogen recovery following molecular analysis.*

5. Place laboratory samples (Container 1) within a BSL-2 hood upon arrival at the laboratory, homogenize by shaking, and level off to consistent volumes (500 mL) prior to pretreatment. (Note: 500 mL collection bottles have room for more volume in the wide-mouth part of the bottle; for grab samples especially, a volume >500 mL may be collected.) Pour any excess wastewater (i.e., volume >500 mL) into a sterile beaker to lower the water line to the level indicated on the pre-labeled bottle to the desired volume (500 mL). Discard the excess wastewater from the sterile beaker once the sample in the bottle is leveled. Autoclave beaker(s) after use.

*Note: A pre-sterilized beaker is necessary in case excess sample is poured out, and some must be returned to the sample bottle to achieve a 500 mL volume. Use a different, sterile beaker per wastewater sample to avoid cross-contaminating samples.*

**Caution:** For all steps within the laboratory while handling wastewater, appropriate PPE is to be worn (lab coat, gloves, disposable face mask, long pants, closed-toed shoes, and tied-back hair). Additional steps of disinfection with 99.5% isopropanol must be taken to ensure that no cross-contamination occurs between samples (i.e., work with one sample at a time, disinfect outside of sample bottle and equipment sample came in contact with, replace gloves, and disinfect any surface/equipment that was contaminated once you are finished), and sterile technique should be used for all processing steps. Before removing anything from the BSL-2 hood, be sure to disinfect using 99.5% isopropanol.

**B. Preparation of biological recovery controls for addition to wastewater**

**Viral recovery control, OC43**

1. For generating the OC43 viral control, Vero cells are grown in a T75 flask to 50% confluency and infected at an MOI 0.01 using a previously frozen aliquot of OC43 virus for 1.5 h in 12 mL of RPMI media at room temperature on a plate rocker.

2. After infection, add 12 mL of RPMI media to the flask and incubate cells at 37 °C overnight.

3. The following morning, remove media by aspiration and immediately replace with new media (12 mL). Incubate cells at 37 °C for four days.

4. Filter culture medium containing viral particles using a Pall 0.45-µm filter unit.

5. Purify total RNA from 50 μL aliquots of virus stock in quadruplicate and quantify by qPCR (absolute quantification alongside standards) to determine genomic copies per reaction of the viral stock.

6. Create aliquots that hold enough viral OC43 RNA to spike a desired amount of wastewater samples, per day of collection, at the desired concentration. Typically, the volume of recovery controls varies between 10 and 100 µL (V_virus _below). This volume is then multiplied by the number of samples expected per day and then by 1.2 to add another 20% volume to the viral aliquot volume to allow for adjustments on the day of sample collection. If the viral RNA spike is too concentrated, it can be diluted using RPMI media.

*Note: To compute the volume of the spike for one sample, the concentration of the OC43 stock solution in genomic copies per microliter (C_virus_) is needed and provided through step B5. If, for example, the goal is to spike a volume of OC43, V_virus_, in microliters, to a set volume of wastewater, V_sample_, in milliliters, to obtain 10^6^ genomic copies per liter of wastewater sample, the equation to determine the spike volume is:*

$$V_{virus}=\frac{10^{6}\times V_{sample}}{1000\times C_{virus}}$$

The 1,000 in the above equation is used to convert from milliliters of wastewater sample to be spiked, *V_sample_*, into units of liters.

7. Using a heat block, heat-inactivate OC43 viral aliquots at 56 °C for 15 min prior to adding to wastewater samples.

8. Add the OC43 recovery control to wastewater at a concentration of 10^6^ genomic copies per liter (gc/L) of wastewater (Graphical overview, Step 1AB). Mix to homogenize. Following the closure of the bottle, spray bottle and surrounding area of the BSL-2 hood with 99.5% isopropanol to disinfect.

**Cell recovery control, *Mycobacterium smegmatis***

9. Transfer *Mycobacterium smegmatis* from glycerol stock onto a LB agar plate containing 100 μg/mL ampicillin (for general selection and to ensure off-target bacteria do not grow, as *M. smegmatis* is resistant to ampicillin). Incubate at 37 °C for three days.

10. Inoculate a single, isolated *M. smegmatis* colony from the fresh plate into LB broth containing 0.05% Tween 80 and 100 μg/mL ampicillin and grow at 37 °C with shaking for 30 h.

11. Harvest cells at mid-log phase as determined by the optical density at 600 nm via analysis with a spectrophotometer (OD_600_ ~1.0 corresponds to a concentration of 1 × 10^8^ cells per milliliter), and estimate the concentration of the *M. smegmatis, C_M.speg_*, in units of genomic copies per microliter (gc/μL).

12. Add *M. smegmatis* recovery control to wastewater at a concentration of 2 × 10^6^ gc/L of wastewater (Graphical overview, Step 1AB). Mix to homogenize. Following the closure of the bottle, spray bottle and surrounding area of the BSL-2 hood with 99.5% isopropanol to disinfect.

*Note: To compute the volume of the spike for one sample, the concentration of the* M. smegmatis *stock solution in genomic copies per microliter (C_M.smeg_) is needed and provided through step B11. If, for example, the goal is to spike a volume of* M. smegmatis, *V_M.smeg_, in microliters, to a set volume of wastewater, V_sample_, in milliliters, to obtain 2 × 10^6^ cells per liter of wastewater sample, the equation to determine the spike volume is:*

$$V_{M.smeg}=\frac{{2\times 10}^{6}\times V_{sample}}{1000\times C_{M.\;smeg}}$$

The 1,000 in the above equation is used to convert from milliliters of wastewater sample to be spiked, *V_sample_*, into units of liters.

**C. Additional pretreatment of wastewater for electronegative membrane filtration**

*Note: This step is recommended for viral RNA recovery.*

1. Vortex the tube containing the biological controls (OC43 and *M. smegmatis*) to homogenize. Add the target volume of the biological control to the sample. Close the bottle and shake to homogenize.

2. Add magnesium chloride to a concentration of 50 mM and an autoclaved magnetic stir bar per sample bottle (Graphical overview, step 1B). If using 51% MgCl_2_, then add 9.4 mL per liter of eluate (= 4.7 mL for 500 mL wastewater sample). Close the bottle and shake to homogenize.

3. Place the wastewater sample bottle on a magnetic stir plate and insert the pre-calibrated (using pH standards 4, 7, and 10) pH probe to record the initial pH of the sample using a pH meter.

4. With the pH probe still inserted, use a sterile glass dropper to adjust the pH of wastewater sample to 3.5–4.5 by adding individual drops of hydrochloric acid (10%), being careful never to touch the dropper to the sample to avoid cross contamination. Record the number of drops used as well as the final resulting pH of the sample from the pH meter (Graphical overview, Step 1B).

5. Close the bottle and shake to homogenize.

**D. Primary concentration by membrane filtration**

1. Attach sterile Pall magnetic filter cup holders securely to the vacuum manifold (ensure that one Pall magnetic filter cup is used per sample to avoid cross-contamination between samples).

2. Remove the magnetic top cup. With a pair of sterile forceps (one that only comes in contact with sterile filter cups; usually kept within a small sterile beaker; autoclave after use), carefully place a MCE membrane filter (if electronegative filtration for both RNA and DNA recovery are chosen; Graphical overview, Step 2B) or GN-6 Metricel membrane filter (vacuum filtration without acid and magnesium chloride pre-treatment if recovering DNA only; Graphical overview; Step 2A) on the base of the filter cup (see General note 2). Reattach magnetic cup, turn on the vacuum, and open the vacuum manifold.

*Note: MCE membrane filters are used for electronegative filtration, where samples have undergone a pH change and addition of salt (MgCl_2_) to help viral particles bind to the filter not only by size but also by electrical charge. Electronegatively charged MCE membrane filters are recommended for viral targets. GN-6 Metricel membrane filters are used for size-selection only and are recommended for use with bacterial and fungal targets for which DNA is extracted. Vacuum filtration with a GN-6 Metricel membrane filter can capture viruses if they adhere to particles larger than 0.45 µm in size. We recommend the preparation of two separate filters, one for RNA extraction (MCE) and another for DNA extraction (GN-6).*

3. Use a sterile, sample-specific graduated cylinder (one per sample, autoclave after use) to measure incremental volumes of wastewater sample to pour into the Pall cup for filtration and concentration of the sample.

**Caution:** Depending on the total number of samples, a lot of equipment goes into successfully performing this procedure. Be sure to use pre-sterilized individual equipment per wastewater sample to avoid cross-contaminating sampling sites and ensure that sterile technique is employed for all sample processing. Pre-label all equipment and supplies that will be in direct contact with samples to avoid cross-contamination.

**Figure 3.**
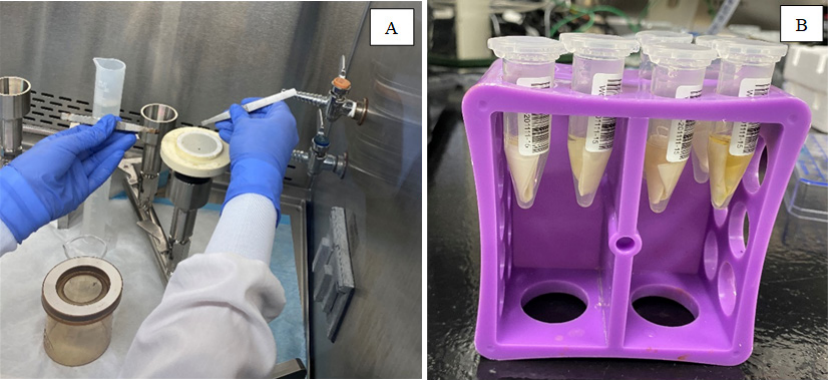
Filter concentration and placement within lysis buffer. After a recorded volume of wastewater is passed through for whole-membrane extraction, the filter is folded into itself three times (A) and then placed into a 5 mL centrifuge tube containing 2 mL of lysis buffer (Zymo DNA/RNA shield) (B).

4. Add wastewater for filtration to the Pall cup (one per sample, autoclave after use) until the filter membrane is completely saturated and minimal flow through the membrane occurs. Record the total volume of wastewater filtered.

5. Remove the magnetic top cup and use a pair of sample-specific forceps (two per sample, autoclave after use) to handle the membrane (Figure 3). Sample-specific forceps should be held within 100 mL beakers to avoid cross-contamination with surfaces, other equipment, or different sample membranes (autoclave after use).

a. For DNA, a half-membrane is to be used for downstream analysis. To prepare the half-membrane, remove the membrane from the filter funnel base and place within a sterile Petri dish. Slice the filter in half with a sterile scalpel, then use sample-specific forceps to fold the membrane in on itself twice. Place half of the folded membrane directly into a 2 mL Zymo ZR BashingBead lysis tube (0.1 & 0.5 mm) containing 1 mL of 1× DNA/RNA Shield (pre-pipetted using sterile technique) and the other half in a second BashingBead lysis tube containing 1 mL of 1× DNA/RNA Shield OR in a 5 mL Eppendorf tube also containing 1 mL of 1× DNA/RNA shield to be used as backup or a technical replicate (Graphical overview, Step 2Ai–ii). The reason a half-membrane is used for downstream DNA analysis is due to the physical size limitations of the BashingBead lysis tubes.

b. For RNA, the whole-membrane is to be used for downstream analysis as the process is not limited by physical size limitations. Carefully fold the membrane in half and then in on itself two more times utilizing the same pair of sample-specific forceps. Place folded membrane directly into a 5 mL Eppendorf tube containing 2 mL of 1× DNA/RNA Shield (pre-pipetted using sterile technique) (Graphical overview, Step 2B).

*Note: When performing RNA analysis workflows only, the volume of 1× DNA/RNA Shield can be reduced to 1.5 mL to increase concentration factors. We recommend the use of 2 mL of 1× DNA/RNA Shield to maintain the consistency of concentrates (1 filter per 2 mL of Shield) between the DNA workflow and the RNA workflow.*

6. Refrigerate wastewater filter concentrates at 4 °C (5 mL Eppendorf tube with full or half membrane or 2 mL Zymo ZR BashingBead lysis tube, 0.1 & 0.5 mm, with half membrane) until ready for further molecular processing. Of note, we do not recommend bead beating prior to RNA extraction as heat/friction may degrade viral nucleic acids plus bead beating releases ribosomal RNA from bacteria and higher microbes, which can interfere with viral RNA. We recommend chemical lysis provided from DNA/RNA Shield followed by slight vortexing for improved viral recoveries.

**Pause point:** The protocol can be paused following the completion of primary concentration, and the sample can be stored at 4 °C until ready for further analysis. We recommend conducting the extractions within 24 h.

7. Rinse and autoclave all equipment used to filter wastewater following its use for individual samples [e.g., Pall cups, forceps, beaker in which forceps were held, graduated cylinders, stir bars (if applicable)].

**E. RNA extraction from wastewater concentrates**

*Note: This step is recommended for the extraction of RNA from viruses.*

1. Prior to molecular analysis, ensure appropriate PPE is used and that benchtop and equipment is decontaminated with 70% ethanol.

2. Following a brief vortex to dislodge and slightly agitate collected solids captured by the membrane (Graphical overview, Step 3), briefly spin down to collect the liquid at the bottom of the tube and pipette 400 μL of wastewater concentrate from the 5 mL Eppendorf tube containing whole membrane into a new, sterile 1.7 mL microcentrifuge tube (Graphical overview, Step 4).

3. Use Zymo Quick-RNA Viral kit for RNA extraction. Add 800 μL of viral RNA buffer (2:1) to the 400 μL sample and mix well by repeated pipetting. All centrifugation steps are performed at 13,000× *g*.

4. Transfer the mixture, 600 μL at a time, over two separate steps, to a Zymo-Spin IC column in a new collection tube. Centrifuge for 2 min. Discard the flowthrough.

5. Add 660 μL of viral wash buffer to the column and centrifuge for 30 s. Discard the flowthrough. Repeat wash step.

6. Add 660 μL of 100% ethanol to the column and centrifuge for 1 min. Discard the flowthrough.

7. Elute the column into a sterile microcentrifuge tube with nuclease-free water.

*Note 1: To compare head-to-head with DNA extraction from wastewater, elute with 100 μL. Continue to step E8 to perform PCR inhibitor removal.*

*Note 2: For standalone RNA analysis from wastewater, elute with 10 μL. Add 30 μL of HIV-1 RNA (approximately 100 copies/μL by qPCR) to the eluted sample to assess PCR inhibition. Alternative inhibition controls can be used as deemed appropriate. Additionally, combine 10 μL of nuclease-free water with 30 μL of HIV-1 RNA to use as a separate no-inhibition control sample. The sample is ready for qPCR analysis (skip steps E7–9).*

8. Optional: Prepare a Zymo-Spin III-HRC column by inserting the column into a new collection tube. Add 600 μL of prep-solution from OneStep PCR Inhibitor Removal kit and centrifuge at 8,000× *g* for 3 min.

9. Place the prepared column into a new 1.7 mL microcentrifuge tube.

10. To remove qPCR inhibitors, transfer 100 μL of RNA eluate into Zymo-Spin III-HRC filters and centrifuge at 16,000× *g* for 3 min.

11. Store samples on ice for immediate analysis by qPCR.

**Pause point:** The protocol can be paused following the completion of RNA extraction, and samples can be stored at -80 °C until ready for further analysis.

**F. DNA extraction from wastewater concentrates**

1. Place 2 mL Zymo ZR BashingBead tubes with half membranes securely into an OMNI Bead-Ruptor 12 instrument and run protocol using the following parameters: 1 min bashing, 5 min rest (3× cycles, 18 min total run time) (Graphical overview, Step 3; see General note 3)

2. Centrifuge 2 mL Zymo ZR BashingBead tube at 12,000× *g* for 1 min to separate particles from solution following bead beating (Graphical overview, Step 4).

3. Transfer 400 μL of supernatant to a Zymo-Spin III-F filter from the ZymoBIOMICS DNA Miniprep kit placed in a clean collection tube and centrifuge at 8,000× *g* for 1 min. Discard the flowthrough.

4. Follow the remainder of ZymoBIOMICS DNA Miniprep kit protocol starting with the addition of 1,200 μL of ZymoBIOMICS DNA binding buffer.

**Pause point:** The protocol can be paused following the completion of the ZymoBIOMICS DNA Miniprep kit, and the sample can be stored at -20 °C until ready for further analysis.

**G. Volcano 2nd Generation qPCR for quantifying RNA targets**

1. Amplify purified RNA directly by qPCR using Volcano2G polymerase in 40 μL reactions with a 4 μL sample input (Graphical overview, Step 5). Prepare a master mix for the number of samples plus an additional 2 according to the following ratios:

a. Nuclease-free water (23.6 μL)

b. 1.1× Volcano buffer (8.8 μL of 5× stock)

c. 200 nM dNTPs (0.8 μL of 10 mM stock)

d. 2 units Volcano2G polymerase (0.4 μL of 5 units/μL)

e. 1 unit anti-Taq antibody (0.2 μL of 5 units/μL)

f. 500 nM target-specific forward primer (Table 1) (1.0 μL of 20 μM stock)

g. 500 nM target-specific reverse primer (Table 1) (1.0 μL of 20 μM stock)

h. 250 nM target-specific probe (FAM or HEX) (Table 1) (0.1 μL of 100 μM stock)

i. 1× Rox (0.1 μL of 400× stock)

**Table 1.** Optimized target-specific primer and probe sequences for V2G-qPCR and qPCR. Primer pairs (f and r) and target-specific probe sequences for all qPCR targets from previous publications utilizing this procedure and approach for analysis.

Molecular target	Primer/probe	Sequence of primer/probe 5′ → 3′
**SARS-CoV-2**	CV3b/f CV3c/r CV3.prb	TGCTAACAAAGACGGCATCA GTAGCACGATTGCAGCATTG ACA TTG GCA CCC GCA ATC CTG CT (FAM)
**Beta-2 Microglobulin**	qB2M/f qB2M/r B2M.prb	CAAGGACTGGTCTTTCTATCTCTTGTAC CTGCTTACATGTCTCGATCCC CAAAGTCACATGGTTCACACGGCAG (FAM or HEX)
**OC43**	OC43/f OC43/r OC43.prb	CAACCAGGCTGATGTCAATAC AAACCTAGTCGGAATAGCCTCA ACATTGTCGATCGGGACCCAAGT (FAM or HEX)
**HIV-1**	RTwt3/f V106/r RT1.prb	GAAAATTAGTAGATTTCAGAGAACTTAATAAGAGAAC CATCACCCACATCCAGTACTGTTA TTCTGGGAAGTTCAATTAGGAATACCACATCCCGCAGG (FAM)
**SIV LTR**	SIV876/f SIV999/r SIV.prb	GCTAGACTCTCACCAGCACTTG CTAGGAGAGATGGGAACACACA TCCACGCTTGCTTGCTTAAAGACCTCT (FAM)
**Mpox**	Mpox/f Mpox/r Mpox.prb	TCTTGCTATCACATAATCTGRAAGCGTA GATATAGCACCACATGCACCA AAGCCGTAATCTATGTTGTCTATCGTGTCC (HEX)
**PMMoV**	PMMoV/f PMMoV/r PMMoV.prb	AGTGGTTTGACCTTAACGTTTGA CCTACGTCTGACGACACAATCT CCTACCGAAGCAAATGTCGCACT (HEX)
***M. smegmatis***	qMsmKat/f qMsmKat/r MsmKat.prb	CCGCTCGAAGAGGTCG GTCCAGGTGACCTCGAGAC TCCTTGCCGACGCCGGTG (HEX)

2. Briefly vortex and centrifuge master mix solution and pipette 36 μL of master mix into individual wells within a 96-well Bio-Rad hard shell plate.

3. For samples, carefully add 4 μL directly into the master mix within each well. For no template controls, add 4 μL of nuclease-free water into the master mix within each well. No-template controls are equivalent to negative controls and should be analyzed for targets to confirm the lack of amplification signal. For standards, add 4 μL of target-specific standards into the master mix spanning five wells on the plate (ranging from 10^1^–10^5^ copies/μL).

4. Seal the plate firmly with Microseal B, remove perforated edges, and spin down the plate in a plate spinner for 15 s to collect liquid at the bottom of the wells.

5. Power up the CFX Connect instrument and specify run parameters per molecular target being analyzed (Tables 2–4).

**Table 2.** Thermocycling conditions for the V2G-qPCR reaction (SARS-CoV-2 and pepper mild mottle virus targets). R^2^ values reported as ≥0.96 and efficiency reported between 95% and 100%.

Step	Temp. (°C)	Duration	No. of cycles
Initial denaturation	88	30 s	1
Denaturation	88	5 s	45
Annealing	T_a_ = 60	20 s	
Extension	72	15 s	

**Table 3.** Thermocycling conditions for the V2G-qPCR reaction (human coronavirus-OC43, HIV, SIV, and Beta-2 microglobulin targets). R^2^ values reported as ≥0.96 and efficiency reported between 95% and 100%.

Step	Temp. (°C)	Duration	No. of cycles
Initial denaturation	88	30 s	1
Denaturation	88	5 s	45
Annealing	T_a_ = 60	15 s	
Extension	72	15 s	

**Table 4.** Thermocycling conditions for the qPCR reaction (Mpox). R^2^ values reported as ≥0.96 and efficiency reported between 95% and 100%.

Step	Temp. (°C)	Duration	No. of cycles
Initial denaturation	95	1 min	1
Denaturation	95	10 s	45
Annealing	T_a_ = 60	20 s	
Extension	72	15 s	

6. Transform raw qPCR values back to gc/L by correcting for qPCR input (4 μL), volume of 1× DNA/RNA shield, and volume of wastewater used to prepare filter concentrate (taking into consideration if a half or full membrane was used). See Data analysis below.

**H. qPCR for quantifying DNA targets**

1. Amplify purified DNA directly by qPCR using 2× TaqMan Fast Universal PCR master mix in 30 μL reactions with a 5 μL sample input (Graphical overview, Step 5).

2. Prepare a master mix for the number of samples plus an additional 2 according to the following ratios:

a. 1× Fast Mix (15 μL of a 2× mix)

b. 500 nM target-specific forward primer (0.75 μL of a 20 μM stock)

c. 500 nM target-specific reverse primers (0.75 μL of a 20 μM stock)

d. 250 nM target-specific probe (0.075 μL of a 100 μM stock)

e. Nuclease-free water (8.5 μL)

3. Briefly vortex and centrifuge master mix solution and pipette 25 μL master mix into individual wells within a 96-well Bio-Rad hard shell plate.

4. For samples, carefully add 5 μL directly into the master mix within each well. For no template controls, add 5 μL of nuclease-free water into the master mix within each well. No template controls are equivalent to negative controls and should be analyzed for targets to confirm the lack of amplification signal. For standards, add 5 μL of target-specific standards into the master mix spanning five wells on the plate (ranging from 10^1^–10^5^ copies/μL).

5. Seal plate firmly with Microseal B, remove perforated edges, and spin down the plate in a plate spinner for 30 s to collect liquid at the bottom of the wells.

6. Power up CFX Connect instrument and specify run parameters per molecular target being analyzed (Table 5).

**Table 5.** Thermocycling conditions for the qPCR reaction (*Mycobacterium smegmatis*)

Step	Temp. (°C)	Duration	No. of cycles
Initial denaturation	95	2 min	1
Denaturation	95	10 s	40
Annealing/Extension	T_a_ = 61	25 s	

7. Transform raw qPCR values back to gc/L by correcting for qPCR input (5 μL), volume of 1× DNA/RNA shield, and volume of wastewater used to prepare filter concentrate (taking into consideration if a half or full membrane was used). See data analysis below.

## Data analysis

To transform the raw qPCR values back to gc/L of raw wastewater, the computations are as follows.

We define the output from the qPCR reactions as genomic copies per reaction (*GCPR*). This is derived from a Cq value that is related, through a standard curve, to genomic copies per reaction. To convert *GCPR* to genomic copies per original volume of wastewater (*GCWW*) in units of genomic copies per liter, the following equation is used:

$$GCWW=\frac{GCPR\times V_{C}\times V_{E}\times 1000}{V_{F}\times V_{A}\times V_{R}}$$

where:

*V_F_* = Volume of wastewater passed through the filter in milliliters (e.g., 60 mL)

*V_C_* = Volume of concentrate in microliters (e.g., 2,000 μL or 1,500 μL volume of DNA/RNA Shield that the folded filter was placed)

*V_A_* = Volume of aliquot of the concentrate used for extraction in microliters (e.g., 400 μL of concentrate was used for extraction).

*V_E_* = Volume of concentrated nucleic acid after extraction in microliters. If additional inhibition controls are added to the *V_E_* (e.g., HIV RNA), the *V_E_* is the sum of the original volume of concentrated nucleic acid plus the volume of liquid added. (e.g., 10 μL of concentrated nucleic acid was produced after extraction and, to this volume, 30 μL of HIV was added for a total *V_E_* of 40 μL of extract).

*V_R_* = Reaction volume used for the PCR reaction in microliters (e.g., 2 μL or 4 μL).

*Note: The 1,000 in the above equation is used to convert V_F_ from milliliters to liters.*

The example calculation utilizing the above numbers follows as:

$$GCWW=\frac{GCPR\times 1500\times 40\times 1000}{60\times 400\times 4}$$

To assess PCR inhibition, quantities of spiked HIV-1 RNA in samples are determined by V2G-qPCR and compared to quantities detected in the control in which HIV-1 RNA was spiked into clean water. A shift to a Cq value higher (±2) than that determined for the water control reveals the magnitude of inhibition that occurs during PCR amplification.

**To compute percent recovery:**

For OC43, assuming an equivalent of 10^6^ gc/L was added to the original wastewater sample, then for each sample for which OC43 was added, compute *GCWW* as above. Percent recovery = (*GCWW* for OC43/10^6^ gc/L) × 100%

For *M. smegmatis*, the percent recovery is computed in a similar fashion; but in this case, 2 × 10^6 ^gc/L of *M. smegmatis* is added to the sample as per the protocol described. As above, compute the *GCWW* for each sample for which *M. smegmatis* was added. Percent recovery = *GCWW* for *M. smegmatis*/(2 × 10^6^ gc/L) × 100%.

**Normalization of raw qPCR data:**

As indicated within Table 1, normalization targets like PMMoV and Beta-2 microglobulin (B2M) have been previously assessed [19,20] utilizing the presented workflows. PMMoV is a virus present in wastewater in high concentrations due to its common presence in human fecal waste, and B2M is a human cellular housekeeping gene that upregulates during inflammation or infection. Our findings indicate that normalization may have benefits at smaller sewersheds but does not help significantly with larger sewersheds. The benefits of normalization will depend upon the variability in the proportion of the wastewater that is represented by human waste relative to the variability of the normalizing measurement itself. We recommend that each researcher evaluate whether normalization is beneficial and utilize a normalization factor applicable to the research to which this protocol is adapted. Additionally, we recommend evaluating several targets’ abilities to provide sound normalization benefits.

*Note: When analyzing data from multiple measurements, statistical tests of normality are recommended to determine if data generated is normal, log-normal, or non-parametric. Statistical tests employed downstream should be appropriate for the intended analysis, specific to the performed experiment, and adjusted for the hypotheses being tested. We recommend analysis with and without outliers, and we also recommend plotting data to further assess outliers as a means of assessing whether statistical tests should include them.*

## Validation of protocol

This protocol has been used and validated in the following research article(s). The filter concentration steps have been validated against ultracentrifugation [22] and against magnetic bead separation [6]. The V2G-qPCR has been validated against traditional RT-qPCR [6,22]. Additional comparisons between different workflows with cross-laboratory comparisons are provided in Zhan et al. [19] and Zhan et al. [21]. The protocol has been validated from detection limits of 10^2^ gc/L to values upward of 10^7^ gc/L. The reproducibility of this proposed workflow was evaluated by Babler et al. [3]. For viral RNA (without bead beating), reproducibility defined by the coefficient of variation was found to be 25% based upon measurements of SARS-CoV-2 and 15% for measurements based upon OC43. For DNA (with bead beating), reproducibility was found to be 14% based upon measurements of *M. smegmatis*. Recoveries (including sample splitting, concentration, lysis, extraction, and qPCR) were 20%, on average based upon measurements of OC43.

• Babler et al. [3]. Expanding a Wastewater-Based Surveillance Methodology for DNA Isolation from a Workflow Optimized for SARS-CoV-2 RNA Quantification. Journal of Biomolecular Techniques. (Figure 1, Figure 2, Table 1).

This protocol in its entirety is based on the publication by Babler et al. 2023; each of the individual steps of electronegative filtration (ENF; filtration with MCE membrane filters), vacuum filtration (VF; using GN-6 Metricel membrane filters), bead beating samples (or excluding), and the separate DNA- and RNA-based extraction methods compare the approaches head-to-head in a detailed manner. Five liters total of wastewater from three sampling sites (one hospital and two from a regional wastewater treatment plant) was collected for this study, where four experimental conditions (ENF with and without bead beating and VF with and without bead beating) were assessed by qPCR downstream. The viral particle spiked-in control, analyzed downstream by qPCR, was human coronavirus-OC43, and the DNA spiked-in control was *Mycobacterium smegmatis.* Figure 1 shows the laboratory workflow performed for analysis, and Figure 2 illustrates the resulting quantification of three molecular targets: SARS-CoV-2, *M. smegmatis*, and OC43. Table 1 provides the specific primers and probes used by qPCR for detecting the molecular targets of focus. Shapiro-Wilk normality tests showed that the majority of the data was normally distributed, and no outliers were observed. As a result, paired t-tests and Pearson correlations were utilized to assess the differences among the methods used. Results of this study showed that the highest recoveries for SARS-CoV-2 corresponded to the VF filter concentrates excluding bead beating, followed closely by the ENF filter concentrates excluding bead beating. Statistical differences were observed between concentrates that underwent bead beating and those that did not with a significant loss in SARS-CoV-2 signal by qPCR with the inclusion of bead beating. For the spiked-in targets, OC43 was similar to SARS-CoV-2 in that bead beating resulted in a significant decrease in RNA detection by qPCR; VF non-bead beat samples resulted in the highest detection by qPCR with a significant difference between the concentrates beat vs. not beat. For the ENF concentrates, bead beat concentrates provided higher recoveries than non-bead beat concentrates. Future research was recommended to further evaluate the differences between the filtration methods. These results validate this protocol by providing replicable results and effective detection utilizing all steps of this listed procedure, including the mentioned controls.

• Babler et al. [14]. Detection of the clinically persistent, pathogenic yeast spp. *Candida auris* from hospital and municipal wastewater in Miami-Dade County, Florida. *Science of the Total Environment* (Figure 2, Table 1).

This publication utilized the vacuum filtration (coupled with GN-6 Metricel membrane filters) portion of this procedure and the following DNA extraction process utilizing the ZymoBIOMICS DNA Miniprep kit coupled with bead beating. Two sampling locations were of primary focus for this manuscript, a hospital housing *C. auris* patients and the corresponding regional wastewater treatment plant. Samples analyzed were part of a weekly surveillance program that spanned from May to September 2022 (38 total 500 mL samples, 19 total weeks from both sites). Routine SARS-CoV-2 data (and patient cases), as a part of the routine surveillance efforts, were also assessed alongside the *C. auris* target to determine if there was a trend between hospital cases of illness and positive signal at both wastewater sites. The *C. auris* target was analyzed by qPCR and compared against positive patient cases admitted to the hospital. No spiked-in controls were utilized for this publication; however, wastewater was separately filtered to culture *C. auris* from wastewater (not mentioned within this protocol), where grown colonies were isolated and analyzed by ClustalW sequence alignment after target amplification and Sanger Sequencing to confirm that they matched the *C. auris* genome described in GenBank at the NCBI. Pearson and Spearman correlation assessments were performed with the data set and compared against clinical case data for infected patients provided by the University’s hospital. Figure 2 illustrates a time series plot containing daily clinical data as well as wastewater signal (detected by qPCR) for *C. auris* as well as SARS-CoV-2. Table 1 provides the specific primers and probes used by qPCR for detecting the molecular targets of focus. Insignificant correlations were illustrated between sampling sites for given weeks and between clinical data and wastewater for these time periods. Future research was recommended based on the results, yet this publication provided valuable data in that *C. auris* was detectable from wastewater utilizing the above methodology and was viable enough for culture using additional methodologies (not described here).

• Sharkey et al. [17]. Monkeypox viral nucleic acids detected using both DNA and RNA extraction workflows. *Science of the Total Environment*. (Figure 2, Figure 3, Table 1).

The entirety of this protocol was also utilized for this publication, in which the electronegative filtration approach (MCE membrane filter not coupled with bead beating) and the VF approach utilizing bead beating were employed. The long-standing WBS program at the University of Miami employed the filtration approach with MCE membrane filters, and for this investigation developed a similar approach of vacuum filtration using GN-6 Metricel membrane filters for the express purpose of detecting DNA pathogens, such as Mpox. Weekly samples from the hospital as well as wastewater treatment plant were utilized spanning the outbreak period in Miami-Dade County of Mpox (June to September 2022). Spiked-in controls, OC43, and *M. smegmatis* were utilized to assess the validity of each workflow using ENF combined with the described RNA extraction and VF combined with the described DNA extraction. Clinical data was also utilized, since this investigation was a response to a public health outbreak with positive patient cases at the hospital samples for wastewater. Figure 2 illustrates the genomic copies per liter (gc/L) of Mpox DNA from wastewater detected by qPCR, and Figure 3 illustrates the gc/L of Mpox RNA, likely attributable to the detection of Mpox RNA being expressed in infected human cells that had been introduced into the wastewater. This study followed a detection-based approach. The correspondence focused on the presence of Mpox within wastewater and evaluated if that aligned with dates of Mpox-positive patients in the hospital. This study validates this listed procedure by providing evidence for the ability to use these methods to detect DNA viruses of concern such as Mpox.

• Babler et al. [11]. Degradation rates influence the ability of composite samples to represent 24-hourly means of SARS-CoV-2 and other microbiological target measures in wastewater. *Science of the Total Environment* (Figure 1, Figure 2, Figure 3).

This work used the MCE membrane filter and electronegative filtration process of this procedure, coupled with the RNA extraction portion, in a thorough investigation of timepoints to assess changes in microbial target concentrations. Three separate experiments, corresponding to a community scale (wastewater treatment plant, WWTP), cluster-of-buildings scale (connected by the same sewer line), and building scale sewershed sampling site, collecting 24 samples for assessment by V2G-qPCR were performed (n = 72 collections, n = 4 composites, three collected and one given by the wastewater treatment plant, “B” set of experiments). Additionally, at the start of each experiment, 16 L of wastewater was collected and split into 24 aliquots to be assessed individually alongside hourly collections from the sewer system (n = 72 aliquots from 3× large collections, “A” set of experiments). The procedure followed in this publication slightly modified the total volume of wastewater collected for necessary splits for assessing water quality, creating a composite from every hourly collected sample, and assessing every hourly sample with filtration. The filtration-based procedure, however, was not modified, and each hourly sample was able to be pretreated and filtered within the allotted time. The OC43 spiked-in control was utilized per sample, with an additional viral control of SIV also being added to assess degradation over time. Figure 1 illustrates sample splitting and analysis performed on the large collection (16 L) performed at the start of each experiment. Figure 2 illustrates sample splitting and analysis performed on the individual collections, taken every hour from the sewer system per sewer shed. As SARS-CoV-2 degradation and stability were the primary focus of this study, Figure 3 illustrates the gc/L of SARS-CoV-2 detected from each hourly sample, the aliquoted hourly assessed sample, and composites created per experiment for each scale of sample collection. Table 1 provides the specific primers and probes used by qPCR for detecting the molecular targets of focus. Shapiro-Wilk normality tests were performed on all qPCR datasets. Non-normal distributions were determined per molecular target assessed (all targets: SARS-CoV-2, SIV, PMMoV, B2M, HIV, and OC43); therefore, Spearman correlation tests were used to evaluate associations between variables over time. Degradation rates were determined per target and per experiment from the slope of the best-fit line between the natural logarithm of the target concentration at hour T divided by the initial target concentration. Moreover, one-sample t-tests were also performed to evaluate whether the mean of the hourly sample (“A” experiments) was statistically different than the created composite for that experiment. For the “B” experiments, one-sample Wilcoxon signed rank tests and one-sample t-tests were also performed to compare means between the grab samples and for the effective comparison of the 24 collected samples with the created composite. Additionally, the homogeneity of variance was evaluated using Levene’s tests for assessing the level of variability between each sewer shed scale from hour to hour. Ultimately, the stability of SARS-CoV-2, B2M, and PMMoV were illustrated by the assessment performed here over the course of 24 h. SIV degraded significantly over the 24-h timeframe, providing evidence that the other targets remained stable. Hourly variability between sewersheds and collected samples further supported that at any point in time, the levels of pathogens found within wastewater can be significantly higher or lower. These results solidify the recommendation of composite sampling to obtain a better estimate of target levels within any given day within a sewer system. The reproducibility of this study and thorough investigation into several different molecular targets validated the long-standing approach used by the University of Miami, described here, which employs pretreatment coupled with MCE membrane filter and electronegative filtration followed by RNA extraction and V2G-qPCR assessment.

• Babler et al. [6]. Comparison of Electronegative Filtration to Magnetic Bead-Based Concentration and V2G-qPCR to RT-qPCR for Quantifying Viral SARS-CoV-2 RNA from Wastewater. *ACS ES&T Water* (Figure 1, Figure 2, Figure 3, Table 2).

The purpose of this publication was to compare the MCE membrane filter coupled with electronegative filtration against another method of primary concentration, utilizing magnetic beads to determine the overall validity and effectiveness of employing filtration-based approaches for WBS. A total of 13 samples (corresponding to 5 total weeks and multiple study sites) were utilized for this experiment, and the RNA extraction process described above, followed by V2G-qPCR and a mainstream RT-qPCR approach, were employed. The additional comparison of V2G-qPCR against RT-qPCR was to validate the qPCR assay, since commercial kits (especially RT-qPCR kits) during the COVID-19 pandemic were difficult to obtain, keep stocked, and used consistently as many labs employed their use. Figure 1 visually illustrates the workflow performed in head-to-head comparison of the two primary concentration methods and qPCR analyses. Figure 2 provides the correlations between the electronegative filtration and magnetic bead-based approaches for SARS-CoV-2, B2M, and OC43, utilized here as a spiked-in control. Furthermore, Figure 3 provides the correlations for three SARS-CoV-2 genes (N3, N1, and ORF1ab) between the two qPCR approaches. Shapiro-Wilk normality assessments were run on each set of data per concentration method and molecular target. Spearman correlations were used to compare the log-transformed viral concentrations between the primary concentration and qPCR methods investigated. Mann-Whitney U tests were also used to evaluate whether the means for the data sets were statistically equivalent. Ultimately, the ability to detect SARS-CoV-2 and B2M was statistically equivalent between the two primary concentration methods, illustrating the use of both methodologies as sound strategies for detecting these molecular targets within wastewater. OC43 was generally better detected by electronegative filtration than by the magnetic bead-based approach, which further validates the use of filtration methodologies with the intended use of inactivated, spiked-in viruses. Between the qPCR approaches, correlations provided statistical equivalency for the V2G-qPCR approach with mainstream RT-qPCR approaches describing the experimental soundness of the assay. This publication validates the procedure listed here as a sound methodology, which is equivalently comparable to another commonly used primary concentration approach (magnetic-bead based). Moreover, this study validates the use of V2G-qPCR as an effective alternative to RT-qPCR for quantifying nucleic acids from wastewater.

## General notes and troubleshooting

**General notes**

1. This protocol did not discuss the processing of DNA viruses. For DNA viruses, use steps in section E but with the ZymoBIOMICS DNA Miniprep kit instead of the Zymo Quick-RNA Viral kit.

2. In some cases, the addition of acid as part of electronegative filtration may contribute to the decay of nucleic acids. If recoveries are low for viruses, and wastewater solids are visible, consider omitting the HCl and MgCl_2_ addition, as there is a possibility that the viruses may have preferentially partitioned toward the solids and can therefore be removed without charge attraction.

3. If the HCl and MgCl_2_ addition is a concern for DNA recovery of bacteria and higher organisms, consider splitting the wastewater sample before the HCl and MgCl_2_ addition step. If DNA viruses are to be also analyzed, we recommend testing to determine whether the HCl and MgCl_2 _addition improves viral DNA recovery.

4. If recoveries are low for viruses, consider alternative concentration methods such as magnetic bead-based technologies or ultracentrifugation methods. These alternative concentration methods may be necessary for samples with very low suspended solids.

5. Biological controls recommended in this protocol to assess overall process recoveries are OC43 for RNA viruses and *M. smegmatis* for microbes with cell walls. Other viral, bacterial, fungal, and protozoa controls can be utilized instead and may be more applicable depending on the target nucleic acids.

6. MCE membrane filters are negatively charged. For electronegative filtration, salts (e.g., magnesium chloride) and acids (e.g., hydrochloric acid) are added to the sample to change the charge of viral particles to positive. These positively charged viral particles are then attracted to the negatively charged MCE membrane filters and thus captured by charge instead of by size. We have compared results both with electronegative filtration using MCE membrane filters along with regular vacuum filtration using GN-6 Metricel membrane filters (without salt and acid addition), and we obtained similar viral recoveries.

7. Both Pall MCE filters and GN-6 Metricel filters have the same pore size (0.45 µm) and both filters can also remove viruses by size exclusion if the they partition toward larger particulates that are common in wastewater. Therefore, GN-7. Metricel filters without acid or MgCl_2_ (as listed in 1 above) may be suitable for virus capture in wastewater. For viruses in dilute suspensions, evaluate the suitability of both filtration methods, as filtration processes even with charge capture (e.g., electronegative filtration), may not be suitable in their capture on a membrane.

8. Regarding the inclusion or exclusion of bead beating, researchers should consider the heat and friction naturally included with the process and determine if there is a positive or negative impact on the downstream yield or quantity of the sample/targets being analyzed. Bead beating or mechanical disruption of complex cell walls is necessary to release nucleic acids from bacteria, fungi, and protozoa. Bead beating may not be necessary for viruses as off-the-shelf lysis solutions such as DNA/RNA shield are capable of chemically lysing most viruses. Bead beating may be counter-productive to viral recoveries in heterogeneous mixtures such as wastewater. Bead beating releases nucleic acids from most microbes in wastewater including highly abundant ribosomal RNA, which may interfere with the ability to detect viral genetic targets [3]. As a result, we do not recommend bead beating if the focus is on analyzing viral targets. Additional experimentation may be necessary for specific matrix and target combinations.

**Troubleshooting**

Problem 1: Low flow in sewer hole, making desired volume of collection difficult.

Possible cause: In these cases, there are specialized sample collection systems, such as cutting off the screw top portion of the 2 L sample collection bottles to allow the bottle to lie flatter and closer to the bottom of the sewer pipe. Alternatively, there are sample collection nozzles that have been designed to capture water in as little as 1 cm of flow depth. These specialized nozzles are generally weighted and create a vacuum with inlets below the 1-cm threshold, allowing for peristaltic pumps of composite samplers to retrieve wastewater.

Solution: If the problem keeps occurring, consider changing the location and/or timing of sampling collection.

Problem 2: pH of wastewater sample difficult to lower with HCl.

Possible cause: Highly buffered sample, which may be impacted by turbidity and has been observed especially when sewers are undergoing construction due to suspected entrainment of calcium carbonate.

Solution: Adjust pH to range appropriately as possible but do not overload the sample with HCl for risk of degrading pathogens of interest. Proceed with a higher pH if necessary and document.

Problem 3: pH of wastewater sample already below the target pH.

Possible cause: Acid added to wastewater at the source.

Solution: Record the pH. Proceed with a lower pH and document.

Problem 4: Very turbid and particulate-filled wastewater sample.

Possible cause: Sampling sites like wastewater treatment plants can have unusually turbid and particulate-filled wastewater samples on any given day, dependent on the activities of the general public, weather patterns, etc.

Solution: When utilizing filtration-based approaches, if a sample is generally darker or more opaque-looking, it is a good rule of thumb to start with a low volume and then gradually increase until the membrane becomes clogged and saturated. Some samples/sampling sites can saturate a membrane with 5–10 mL when that site normally would saturate at 30–70 mL.

Problem 5: Very clear and translucent wastewater sample.

Possible cause: For sampling sites that do not receive much waste from households or that are close to bodies of water (i.e., lakes and oceans), wastewater samples can look no different from non-potable water.

Solution: Samples that are exceptionally clear and do not visually look like wastewater require higher volumes to saturate membranes. Volumes up to 150–200 mL need to be filtered to appropriately saturate a membrane.

Problem 6: Low recoveries of nucleic acid following bead beating of membranes.

Possible cause: Unoptimized membrane size where tube/beads cannot rupture membrane enough, or bashing protocol generating too much heat, or friction, which can degrade pathogens in wastewater.

Solution: Consider testing different cycling parameters for each specific bead-beating machine and perform multiple head-to-head comparisons of sample yield. If a membrane is too destroyed (e.g., powder-like), it can have a negative impact on yield, similarly to if the membrane is not shredded enough (e.g., large membrane particles/chunks) where particulate is not released from the membrane into solution.

Problem 7: Inhibition detected from the inhibition control.

Possible cause: The chemical make-up of the sample is interfering with the PCR process.

Solution: Consider running dilutions of the sample in an attempt to dilute out the inhibitors. Alternatively, consider integrating inhibitor clean-up (e.g., OneStep PCR Inhibitor Removal kit, as described in step E8). Also, adjusting the elution volume from nucleic acid extraction columns may also help to overcome inhibition (see Sharkey et al. [22] for details).

Problem 8: Negative values for target.

Possible cause: Could be a false negative or truly a negative.

Solution: Check if positive controls OC43 and *M. smegmatis* were recovered at anticipated levels. Also, check for the PMMoV and B2M normalization targets, which should be present in wastewater with human fecal inputs.
